# The Medical Chronicle: A Monthly Record of the Progress of the Medical Sciences

**Published:** 1884-12

**Authors:** 


*The Medical Chronicle: A Monthly Record of the Progress of the Medical Sciences.* Vol. I. Nos. I. & II. Pp. 120. Manchester: J. E. CORNISH. October and November, 1884.

We heartily welcome this most recent evidence of the vigour of thought and work in the provinces. If the *Medical Chronicle* can maintain, even approximately, the high promise of its first numbers, it is evident that it must hold a foremost place among British journals; and if we may judge from the names which appear among its supporters and contributors, we can scarcely doubt that it will easily do this.

It is edited by Drs. Niven and Sinclair, and published under the direction of a committee of nine of the most eminent medical men in Manchester. Original articles occupy about one-fourth of the volume; the rest of the space is filled with very full abstracts and occasional criticisms of current medical literature in Europe. The thoroughness and completeness of these abstracts are certainly nowhere surpassed in British medical journalism, and must have entailed a great deal of labour on the part of those who supplied them. The paper and general get-up are admirable, only we *should* like to have its margins cut. We trust that our contemporary may meet with the success its high excellence undoubtedly merits.

